# HIGH RESOLUTION SPATIAL PROTEOGENOMICS TO ASSESS TARGET ENGAGEMENT AND COMPARE SENOLYTIC EFFICACY IN THE BRAIN OF TAU TRANSGENIC MICE

**DOI:** 10.1093/geroni/igae098.1144

**Published:** 2024-12-31

**Authors:** Miranda Orr, Valentina Garbarino, Timothy Orr

**Affiliations:** Wake Forest University School of Medicine, Winston-Salem, North Carolina, United States; UT Health San Antonio, San Antonio, Texas, United States; Wake Forest University School of Medicine

## Abstract

Tau protein accumulation induces cellular senescence in Alzheimer’s disease and other tauopathies contributing to dysfunction and disability. Treating tau transgenic mice with pharmacological agents to clear senescent cells, “senolytics,” reduced brain pathology and improved structure and function. We now report results from directly comparing the previously used senolytic therapy, dasatinib plus quercetin (D+Q), to fisetin (FIS), a senolytic with a potentially favorable side-effect profile. Fourteen-month-old male and female wild type (WT) and tau transgenic (rTg4510) mice were treated intermittently for 12 weeks with D+Q, FIS or vehicle by oral gavage. Physical and behavioral outcomes were measured before and after treatment, and we utilized spatial proteogenomics for high-resolution target engagement. In vivo assessments included assays for exploratory behavior, cognition, stereotypic behavior, physical performance and frailty. Genotype- and sex-specific treatment effects will be discussed. Both senolytic treatments were equally protective against neurodegeneration as evidenced by smaller lateral ventricle size in senolytic treated rTg4510s compared to those vehicle treated. GeoMx digital spatial profiling and CosMx spatial molecular imaging data indicate treatment-specific effects with a genotype interaction. In general D+Q exerted a greater effect on tau neuropathology in rTg4510 mice; other treatment-specific differences across cell types and brain regions will be presented. Overall our data suggest that tailoring senolytic therapies to specific individuals in the future based on sex and disease state may provide the most benefit as we continue to translate this promising therapy to clinical use.

